# Patient safety incidents in paediatric radiology: how to care for the professional?

**DOI:** 10.1007/s00247-024-06054-9

**Published:** 2024-09-18

**Authors:** Rutger A. J. Nievelstein, Marije P. Hennus, Marjel van Dam

**Affiliations:** 1 Department of Paediatric Radiology & Nuclear Medicine, Division of Imaging & Oncology, University Medical Centre Utrecht/Wilhelmina Children’s Hospital, P.O. Box 85500, 3508 GA Utrecht, The Netherlands; 2 Department of Paediatric Intensive Care Medicine, Division of Paediatrics, University Medical Centre Utrecht/Wilhelmina Children’s Hospital, Utrecht, The Netherlands; 3 Intensive Care Centre, Division of Vital Functions, University Medical Centre Utrecht, Utrecht, The Netherlands

**Keywords:** Coping strategies, Incidents, Paediatric radiology, Patient safety, Radiology

## Abstract

Patient safety incidents in paediatric radiology have profound impacts not only on the patient and their family, but also on the well-being and professional practice of healthcare professionals. These incidents, which range from procedural and diagnostic incidents to serious adverse events leading to harm or even death of the patient, may evoke feelings of distress, guilt, and anxiety among paediatric radiologists (in-training), ultimately affecting their confidence and ability to deliver high-quality care. Recognizing the importance of addressing these challenges, healthcare organizations should implement strategies to support professionals in coping with and learning from these incidents. By fostering a culture of open communication, providing access to peer support, and offering structured debriefing and educational opportunities, healthcare institutions can help mitigate the psychological toll of patient safety incidents and promote resilience among their staff. This article examines the multifaceted impacts of patient safety incidents on paediatric radiologists (in-training) and their staff and outlines effective approaches for handling these incidents to support professional well-being and enhance patient safety.

**Graphical abstract:**

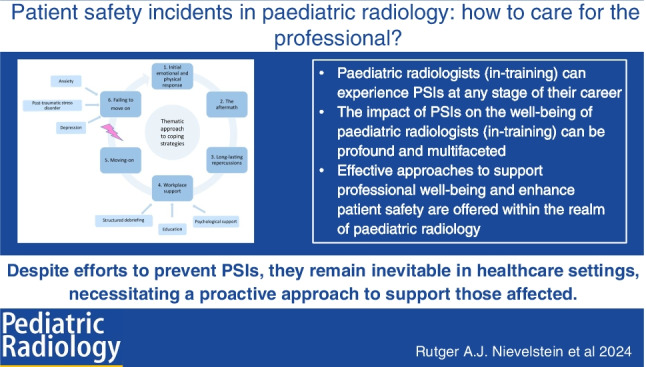

## Introduction

Patient safety incidents (PSIs) are events or circumstances that could lead to, or have led to, unnecessary harm to a patient [1]. In paediatric radiology, these incidents can occur during diagnostic and image-guided interventional procedures, posing significant risks to patient safety and the quality of care. PSIs in paediatric radiology can include procedure-related issues like radiation exposure, technical errors, or contrast media reactions, as well as diagnostic mistakes, communication failures, and external factors such as logistical challenges or inconsistent regulations [2, 3]. Additionally, patient-related incidents, such as identification errors or breakdowns in communication with patients and families, can also occur. Serious adverse events (SAEs) are a subset of PSIs that result in significant harm to the patient [4], including severe allergic reactions, excessive radiation exposure, or life-threatening complications during procedures.

Research indicates that up to half of healthcare professionals experience PSIs at least once during their careers [5, 6]. Although research specific to (paediatric) radiology is limited, the relevance and impact of these incidents are undeniable, underscoring the need for heightened awareness and strategies to address them. The impact of PSIs, namely, may extend beyond the immediate harm to patients, profoundly affecting the well-being and performance of healthcare professionals involved, including paediatric radiologists (in-training) [6–10]. These incidents can lead to emotional distress, feelings of guilt, anxiety, decreased job satisfaction, and an increased risk of burnout and depression. The term “second victim”, introduced by Albert Wu in a BMJ editorial [11], highlights the emotional toll on healthcare workers who are involved in unanticipated adverse events or errors [12]. The initial purpose of Wu was to bring attention to the need of emotional support for healthcare professionals who have been involved in a SAE. Although the term has been adopted by the literature since then, there is also debate about whether it is inappropriate to use. Several researchers argue that its use might suggest that healthcare professionals are avoiding their responsibility for patient safety and impact of SAEs on patients and their loved ones [13–15].

Perception of seriousness of PSIs can vary among professionals and is influenced by factors like vulnerability and resilience to stress-related issues during or after the PSI. Numerous factors, both at work and in the personal situation, determine a person’s vulnerability and resilience, and these factors can change over time [16]. Work-related factors include work demands (organization and responsibilities), work schedule, job satisfaction (quality of work), working atmosphere (intercollegiate contact, including with managers and supervisors), and work-life balance. On the other hand, personal life events (such as serious illness or death of a loved one, but also keeping family life going) may impact a healthcare professional’s ability to cope.

Interestingly, there is often an assumption that healthcare professionals can deal with PSIs, independent of their severity and seriousness, and return to adequate cognitive and emotional function shortly afterwards to continue their professional responsibilities [17, 18]. However, the aforementioned emotional toll underscores the importance of comprehensive support systems within healthcare organizations, including debriefing and counselling, as well as implementation of systematic approaches to incident prevention and learning. This article explores the multifaceted impact of PSI, offering effective approaches to support professional well-being and enhance patient safety within the realm of paediatric radiology.

## Examples of patient safety incidents and their impact

To help set the scene, the potential impacts of two PSIs are described.

**Table:** 

*Case 1*
The first case study describes a PSI during a fluoroscopy-guided procedure in a young child, 8 months of age. The child presented in the children’s hospital during evening hours with fever and malaise during the past week, and episodes of colic abdominal pain, vomiting, and bloody diarrhoea over the last 24 h. The paediatric radiologist on call performed an ultrasonography (US) of the abdomen which showed an ileocolic intussusception. Therefore, the child was referred to the fluoroscopy room for hydrostatic reduction according to protocol including monitoring of vital functions. During the procedure, the child deteriorated clinically and ended up in a resuscitation setting. Direct fluoroscopic and US evaluation showed a partial reduction of the ileocolic intussusception and no signs of procedure-related complications (e.g. perforation). Initially, the resuscitation efforts were effective, and the child was transported to the intensive care unit (ICU) for further stabilization. Unfortunately, shortly after arrival at the ICU, the child ended up in recurrent resuscitation settings and ultimately died due to a severe therapy-resistant septic shock. At the time of this event, workplace support was not as developed as nowadays. Although a short debriefing session shortly after the SAE with all healthcare professionals involved did take place, and the paediatric radiologist did share her experiences and emotions with her colleagues in the days following the event, no further support was offered. Initially, the paediatric radiologist rationalized that (as known from the literature) there is always a small but non-negligible risk of such a complication related to the hydrostatic reduction of an ileocolic intussusception. However, in the following weeks, she began questioning whether her actions and decisions during the hydrostatic reduction might have influenced the outcome, resulting in feelings of guilt and self-blame. In addition, she became afraid of new cases with an intussusception that would require hydrostatic reduction and increasingly tried to avoid triggering situations at the fluoroscopy room. Only after seeking professional support several months later did these emotions subside, and she managed to move on.

**Table:** 

*Case 2*
In this second case study, we present a challenging resuscitation event at an emergency department. A paediatric radiologist was performing an ultrasound on a child during which the child suddenly deteriorated and ended up in a resuscitation setting. The paediatric radiologist immediately started the resuscitation together with one of the nurses until arrival of the resuscitation team, but the family’s emotional distress quickly escalated. Despite efforts to continue resuscitation, one family member began filming, disrupting proceedings. A nurse instructed them to stop, but they remained disruptive. Another family member’s agitation persisted, hindering the resuscitation process, leading to their removal from the room. After 30 min, the resuscitation team unanimously agreed to cease resuscitation due to futility. The paediatric radiologist together with the intensivist leading the resuscitation team informed the family and soon afterwards the resuscitation team terminated the procedure, prompting immediate escalation. One family member, wielding scissors, threatened to take the life of the paediatric radiologist, while another snatched his hospital badge for identification purposes, threatening consequences if the resuscitation efforts were not continued. Multiple de-escalation efforts by the paediatric radiologist and intensivist failed, eventually necessitating the withdrawal of the medical team and intervention by law enforcement. Hours after the death of the patient, the situation eventually calmed down enough for other healthcare professionals to assist the family in the post-mortem care of the patient. Immediately following the event, a team debriefing session was conducted with all healthcare professionals involved addressing technical and situational aspects of the resuscitation, as is customary. Everyone then proceeded with their shift, fuelled by adrenaline, seemingly unaffected. Hours later, as the shift concluded, the institution organized an impromptu, more comprehensive group debriefing session, facilitated by a psychologist, to delve into the exceptional circumstances faced. Individual and group peer support, along with subsequent follow-up sessions, were made available to all healthcare professionals involved. Initially, the involved paediatric radiologist rationalized that such extreme events were unfortunately part of the job. However, once the adrenaline subsided, feelings of anger, anxiety, and insecurity emerged, both at work and at home. It took several weeks and peer support sessions for these emotions to dissipate, as he grappled with concerns not only for his own safety but also for that of his family. Nowadays, he has fully moved on and continues to share this experience during lectures to underscore the intense situations healthcare professionals may have to deal with. During these lectures, he emphasizes that “it’s okay to not to be okay for a while” and encourages openness and seeking help, as it truly makes a difference.

## Thematic approach to coping strategies

**Fig. 1:**
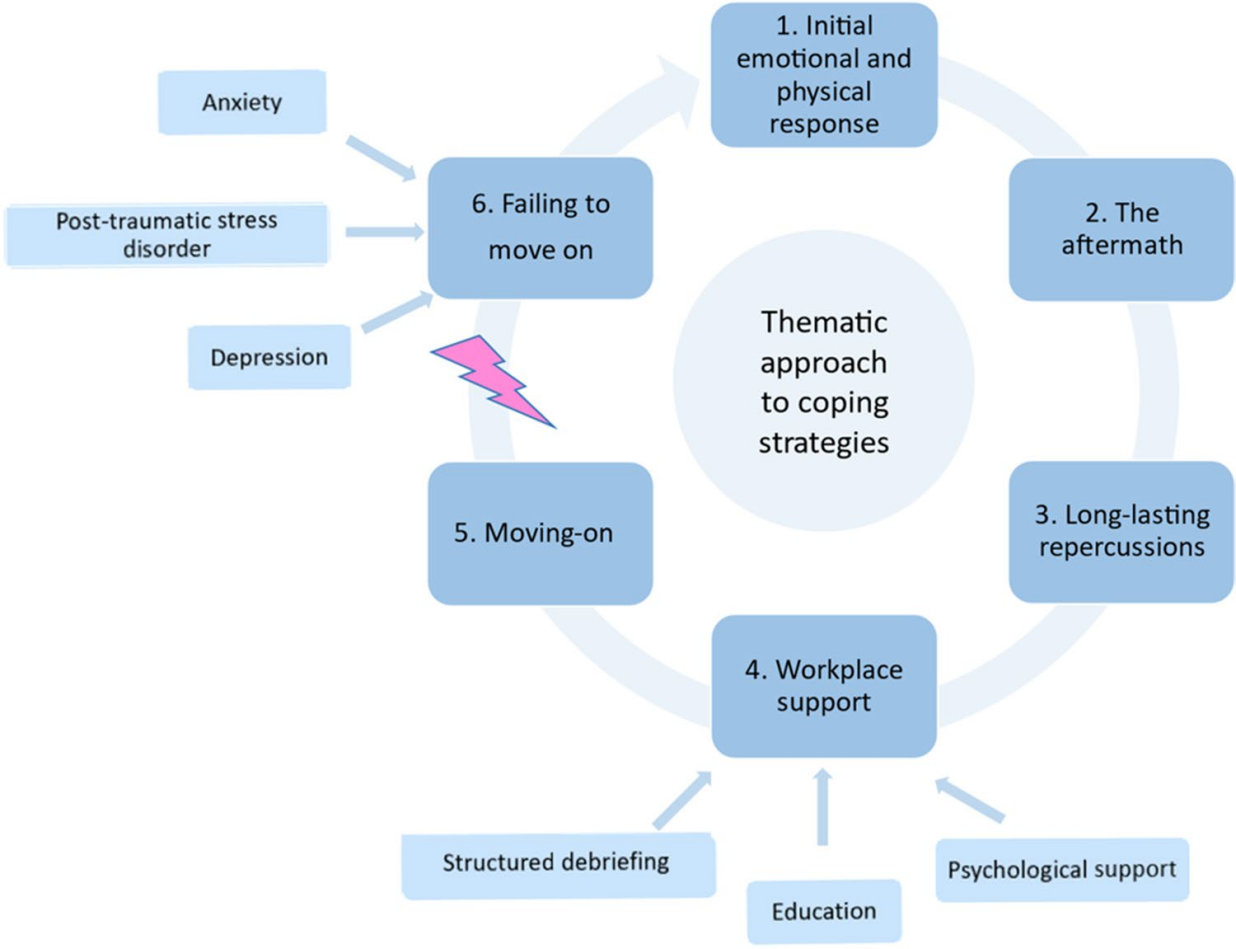
Schematic presentation of the thematic approach to coping strategies and potential long-term posttraumatic symptoms after a patient safety incident

Recovery from a PSI perceived as serious is known to take weeks or even months, and social support as well as active problem-focused coping can help in handling traumatic stressors and avoiding long-term emotional dysregulation. Based on a recent study by Buhlmann et al. [7] in nurses and midwives, five main themes can be distinguished in the period following a PSI perceived as serious. As failing to move on after a PSI does also happen, even when preventive measures are offered, this topic is added to this thematic approach (Fig. 1):

1. Initial emotional and physical response
2. The aftermath
3. Long-lasting repercussions
4. Workplace support
5. Moving-on
6. Failing to move on

The themes will now be discussed in more detail based on current literature as well as our own experience. Furthermore, potential strategies to deal with the themes will be provided to minimize the risk of developing long-term post-traumatic stress symptoms, anxiety, and depression.

1. *Initial emotional and physical response*
  When involved in a PSI perceived as serious, healthcare professionals often experience a range of emotional and physical responses [19]. Immediately after the incident, this may include shock, soon followed by feelings of stress and disbelief. Nevertheless, by relying on their instincts and switching to an “autopilot mode,” the professional often still manages to keep functioning in the early phase after the incident. These emotional or psychological responses are often accompanied by physical reactions as increased heart rate, blood pressure, and muscle tension. After this initial phase, feelings of guilt and self-blame may develop, as professionals question their actions and decisions during the incident, feeling responsible for the outcome. Other emotional responses include heightened levels of fear, worry, or apprehension about the incident and its consequences, feeling angry or frustrated about the situation or perceived failure in patient care. Additionally, feelings of powerlessness, sadness, and loss of self-esteem may arise.
  Research suggests, regardless of the nature of the incident, that sharing these initial feelings with someone who understands the experience is an invaluable coping strategy [19–24]. Informal discussions with colleagues, within the (paediatric) radiology department for instance, can provide reassurance, validation, and a more realistic perspective on the incident. Compassion from family and friends can also be very helpful in this phase.
2. *The aftermath*
  The aftermath refers to the first days to weeks after a PSI perceived as serious. During this period, shortly after initial reactions have stabilized, healthcare professionals search for ways to reduce the impact and try to resume normalcy in their work and personal lives. This phase is characterized by repeated experience of the incident in the mind, repeated self-evaluation, apprehension about potential investigations, and concerns regarding job repercussions [7, 17]. Coping with these reflective thoughts is often challenging and will affect job performance and professional practice, potentially leading to avoidance of triggering situations and even reduced clinical work hours, as illustrated by the first case [17]. In addition, there is an increased risk of decreased job satisfaction, compassion fatigue, and burnout.
  Research suggests that healthcare professionals often struggle to cope independently and even though they feel compelled to seek help, they often delay initiating it [7]. Reasons for this delay vary and include not only thoughts that they are expected to handle the PSI themselves as standard part of their job, but also expectations that their immediate supervisor/manager or healthcare organization will contact them shortly after the incident to offer supportive care. However, according to a study by Mayer et al. [19], this rarely occurred. Therefore, addressing the aftermath requires a comprehensive approach that includes debriefing sessions shortly after the incident with all healthcare professionals involved, actively offering individual or group peer support as well as access to other mental health resources.
3. *Long-lasting repercussions*
  This theme refers to the duration of emotional (and sometimes physical) feelings that, although most severe immediately following a PSI perceived as serious, can last for a prolonged period and still be present years later. Moreover, a higher degree of harm may lead to a longer duration of the symptoms [25]. PSIs, and SAEs in particular, have the potential of becoming a life-changing event that will form a permanent imprint in the memory of those involved [26]. These long-lasting feelings and memories may affect not only work performance but also personal lives.
  Not surprisingly, the level of support from the workplace and/or healthcare organization plays a pivotal role in the severity and occurrence of these long-term complaints [7]. Although defensive coping may offer short-term relief, it can hinder full recovery and increase the risk of post-traumatic stress symptoms (s.a. intrusions, avoidance, and hyper-arousal), and even post-traumatic stress disorder (PTSD) [27]. Studies report that approximately 30% of healthcare professionals experience post-traumatic stress symptoms after a PSI perceived as serious, with around 12% developing PTSD [28, 29]. Nevertheless, relatively few healthcare professionals seek help even though they feel impaired in one or more important areas of functioning [28]. They often seem to underestimate the impact of the incident on their personal and working life, and still fear that seeking help would indicate inadequate coping skills, inadequate ability to handle responsibilities, and/or a loss of reputation. A meta-analysis by de Boer et al. [8] has demonstrated, however, that PSIs perceived as serious are positively related to post-traumatic stress symptoms, anxiety, and depression in hospital-based health professionals, and that the effect was more pronounced in the longer than shorter term. They, therefore, conclude that healthcare professionals and their supervisors should be aware of the harmful effects of a PSI and should take preventive measures where possible.
4. *Workplace support*
  As evident from the previous themes, adequate workplace support stands out as one of the most important strategies to mitigate the impact of a PSI on the mental health of healthcare professionals and risk of developing long-term post-traumatic stress symptoms [30]. This starts with fostering a positive “no blame” workplace culture in which emotional and professional recovery after an event is facilitated and normalized. An essential part of this culture is the immediate personal attention of the supervisor or manager shortly after the incident with focus on how the professional is coping and what the needs are for support. Secondly, one or more debriefing sessions with all the healthcare professionals involved can be very beneficial, especially when there is room for the personal and emotional experiences and needs. Easy access to peer support has also been described in the literature as a crucial factor in coping with the multi-faceted impact of a PSI [31].
  However, literature indicates that the workplace support often falls short, with healthcare professionals frequently perceiving the assistance received as inadequate [32, 33]. Despite efforts by healthcare organizations, the support offered may not fully address the real problem or cater to individual needs. Debriefing sessions, if solely focused on risk management and clinical improvement while neglecting personal and emotional needs, can be counterproductive and threaten resilience and recovery [34]. Continuous evaluation and implementation of appropriately designed support and intervention programs are therefore required, which should always include options for personalized assistance.
  In summary, based on the literature in other disciplines and medical specialities, the following preventive measures can be considered as workplace support to reduce the risk of developing post-traumatic stress symptoms and PTSD after a PSI, and improve the mental health and resilience of paediatric radiologists (in-training) [16, 35–39]:
  1. Creating a supportive work environment, where all paediatric radiologists (in-training) and their staff involved in patient care feel valued, respected, and encouraged to seek help when needed. This might also include normalizing seeking help, regular check-ins to monitor their well-being, and providing opportunities to express concerns or seek assistance.
  2. Organizing structured debriefing sessions after a PSI perceived as serious, which can help the paediatric radiologists (in-training) and their staff discuss their experiences, emotions, and reactions in a supportive environment.
  3. Providing access to psychological support including individual counselling, peer support groups, and, if indicated, therapy.
  4. Offering education and training programs on stress management, resilience-building, and coping strategies. This can equip paediatric radiologists (in-training) and their staff with tools to deal with challenging situations in daily healthcare.
5. *Moving-on*
  The ability of a healthcare professional to move on mainly depends on factors such as individual resilience, social and workspace support, professional growth, and self-care practices. Several studies have shown that a higher level of resilience will strengthen the individual’s ability to cope with the impact of a PSI perceived as serious and move on after this experience [40, 41]. Furthermore, actively seeking social and peer support, as well as engaging in self-reflection, positively influences the process of moving on and resuming the professional activities [42, 43]. On the other hand, a study by Mira et al. [44] showed that some individuals experienced personal growth and professional development because of what they learned from the incident, which contributed to their ability to move on. In addition, sharing the experiences after a PSI perceived as serious with the direct colleagues, as well as organizational learning and improvement initiatives, can help the healthcare professional find meaning in their experiences and facilitate moving on. This is nicely illustrated in the second case. Finally, the importance of self-care and well-being practices for healthcare professionals should not be underestimated [40, 41, 43]. This includes balancing work and personal life, and mindfulness training and interventions, as well as physical activity and exercise.
6. *Failure to move on*
  Despite efforts to cope and resume normalcy, some individuals find themselves unable to fully overcome the emotional and psychological impact of a PSI perceived as serious. This failure to move on can manifest as persistent feelings of distress, anxiety, or even post-traumatic stress symptoms. Paediatric radiologists (in-training) may continue to experience intrusive thoughts or memories related to the incident, hindering their ability to focus on work or personal life. Additionally, the inability to move on may lead to challenges in professional relationships, job satisfaction, and overall well-being. Addressing this theme requires targeted interventions and support systems tailored to the individual needs of the paediatric radiologist (in-training) concerned to facilitate his/her healing and recovery process. Often, these needs surpass the available support systems of a healthcare institution, necessitating broader societal awareness and resources such as professional mental help services.

## Discussion

This article examines different strategies for supporting the well-being of paediatric radiologists (in-training) following patient safety incidents (PSIs). Such incidents can have a significant impact not only on patients and their families, but also on the well-being and professional practice of healthcare providers (in-training). Addressing PSIs effectively is crucial and, based on the literature, six key themes related to the period following a PSI were discussed, along with suggestions for support measures. These themes include the initial emotional and physical response, the aftermath, potential long-lasting repercussions, the importance of workplace support, the process of moving on, and the challenges of failing to move on.

### Learning from patient safety incidents

Despite their potential severe impact, PSIs provide valuable opportunities for learning and improvement across various dimensions of healthcare related to quality of care, patient safety, and professional well-being [45–47]. For this, an environment where paediatric radiologists (in-training) and their staff feel comfortable to report these incidents and concerns without fear of reprisal is essential. Sharing personal experiences and lessons learned, in addition to root cause and human factor analysis of PSIs, can help implement system changes and interventions. This should involve not only redesigning healthcare processes, implementing checklists, and improving communication protocols, but also debriefing protocols, counselling services, and peer support programs [48]. Furthermore, regular communication of lessons learned, both from PSI analyses and personal (supportive) experiences, will foster a culture of continuous improvement, patient safety, and promoting professional well-being. Lastly, by involving patients and families in the analysis and improvement processes, insights into their perspectives and experiences can be developed. To improve the quality of care, it can be helpful to identify patient safety risks, improve communication, and enhance patient-centred care.

### Practical tips for paediatric radiologists (in-training)

Paediatric radiologists (in-training) can experience a PSI at any stage of their career. Although literature on handling PSIs within (paediatric) radiology is scarce, much of the knowledge from research and experiences in other disciplines are applicable within the paediatric radiology context. This highlights a significant blind spot in our (sub)speciality that needs more attention. To provide scaffolding in not only coping with but also moving on and learning from these events, we propose the following checklist for paediatric radiologists (in-training) (Table 1).

**Table 1:** Checklist for paediatric radiologists (in-training) after experiencing a patient safety incident

“What to do after a PSI” checklist for paediatric radiologists (in-training)
1. Acknowledge that feelings such as self-doubt, shame, anger, loss of confidence, and other manifestations of grief are normal after a PSI
2. Share those feelings with your colleagues, and if possible, with family and/or friends
3. A debriefing session with all involved healthcare professionals shortly after the incident helps to address the emotional toll of the PSI
4. Peer support can be helpful after a PSI
5. Mindfulness or other relaxation techniques may help to manage stress symptoms after a PSI
6. Share your experiences with other healthcare professionals within/outside your department and establish a positive “no blame” workplace culture
7. Seek professional support if the aftermath of the PSI does not diminish

*PSI* patient safety incident

## Conclusion

In conclusion, the impact of PSIs on the well-being of paediatric radiologists (in-training) can be profound and multifaceted, affecting both their personal and professional lives. Despite efforts to prevent and mitigate such incidents, they remain inevitable in healthcare settings, necessitating a proactive approach to support those affected. The thematic approach outlined in this article highlights the complex nature of coping strategies following PSIs, emphasizing the importance of timely interventions and comprehensive support systems. Furthermore, a checklist “What to do after a PSI” for paediatric radiologists (in-training) is provided.
